# Dietary Protein Hydrolysate From *Calanus finmarchicus* Reduces Oxidative Stress and Increases Intestinal Health in European Sea Bass (*Dicentrarchus labrax*) Juveniles

**DOI:** 10.1155/anu/5531437

**Published:** 2025-08-06

**Authors:** Isak Bøgwald, Alice Marie Pedersen, Jorge Dias, Sileshi Gizachew Wubshet, Karl-Erik Eilertsen

**Affiliations:** ^1^The Norwegian College of Fishery Science, UIT – The Arctic University of Norway, Tromsø, Norway; ^2^Calanus AS, Tromsø, Norway; ^3^SPAROS Lda, Olhão, Portugal; ^4^Nofima AS – Norwegian Institute of Food, Fisheries and Aquaculture Research, Ås, Norway

## Abstract

The aquaculture industry is in dire need of novel feed ingredients that can improve the health and welfare of farmed fish and shrimp. Zooplankton are natural and underutilized marine resources with the potential to be part of a nutritional solution. The aim of this study was to determine the health effects for European sea bass juveniles fed diets with a protein hydrolysate from the zooplankton species *Calanus finmarchicus*, a novel raw material for feed ingredients. Calanus hydrolysate (CH) was benchmarked using fish hydrolysates as controls at inclusion rates of 3%–4%, depending on the protein content for each of the hydrolysates to allow equivalent protein levels in the diets. The initial feeding trial was 84 days, with an additional week to perform an inflammatory challenge with a UV-inactivated bacterium. Fish receiving diets with CH inclusion were associated with lower levels of hepatic protein carbonyls, a lower level of calprotectin and higher levels of mucins compared to the control hydrolysate diets. No statistically significant differences among the diets for the biomarkers related to the inflammatory challenge were observed. The study showed that dietary inclusion of CH has the potential to reduce oxidative stress and increase intestinal health, thus, improving the health of European sea bass juveniles. These health-promoting effects, combined with the sustainable origin of *C. finmarchicus* highlight the potential of CH as a novel functional ingredient for future aquaculture feeds. Its use could contribute to reduced reliance on traditional fishmeal sources, thereby, supporting more sustainable and resilient aquaculture practices.

## 1. Introduction

The health and welfare of the fish we farm, are of both ethical and economic concern. Aquaculture is an industry that has increased its production volumes considerably in recent decades, and the intense production systems that have been developed may involve decreased environmental quality and increased stocking density, leading to more production-related diseases and losses [1]. In an industry that produced aquatic animals for a total value of USD 265 billion in 2020 [2], mortalities of 10%–20% for species such as European sea bass (*Dicentrarchus labrax*) and gilthead seabream (*Sparus aurata*) add up to significant losses [3]. Some of these losses are due to natural causes, others could be avoided or reduced significantly by optimizing aspects of the production process. Ingredients of marine origin are still viewed as the best option for securing optimal growth and health of aquatic animals, as the plant-based alternatives have been shown to comprise less favorable nutritive profiles [4]. Due to volume limitations of available marine ingredients, the bulk protein fractions of most feeds now consist of plant proteins, a trend set to continue until a novel solution arises. For fish fed diets containing high levels of plant proteins, functional ingredients are often included in the feeds to secure health, growth, and well-being [5]. Functional ingredients are nonmedicinal feed components, usually classified as nutrients, vitamins, or supplements. Protein hydrolysates can serve as both nutritional and functional ingredients, and their inclusion in fish feeds has been shown to improve growth rates and feed efficiency and to positively affect several health indicators in fish [6, 7]. When determining the performance of feeds in a trial, outcome-based data of anatomy and physiology are often the most relevant. This includes operational indicators of feed intake, growth, and mortality, and laboratory welfare indicators gained from blood, tissue, and organ samples [8]. Biomarkers from biological samples can serve as indicators for immune function, inflammatory response, intestinal health, oxidative stress, metabolism, and so on [9, 10], and reveal information about health status and robustness against disease and adverse environmental changes that can arise in aquaculture settings.

The novelty of this study is the source of a new protein hydrolysate feed ingredient, made from the raw material of the zooplankton species *Calanus finmarchicus*. This copepod serves as a natural food source during the life cycle of most aquatic animals in the Northern Atlantic Ocean. *C. finmarchicus* has an annual production estimated at 290 million tonnes in the Norwegian Sea, where the Norwegian government regulates the commercial harvesting regime [11–13]. The great potential of *C. finmarchicus* for feed applications lies in its substantial annual volumes and natural composition of marine nutrients, making it a significant and sustainable resource for promising functional ingredients for optimal growth and health of farmed fish. As aquaculture intensifies and the supply of traditional marine ingredients such as fishmeal becomes increasingly constrained, novel protein sources with both nutritional and health-promoting properties are essential. Hydrolysate from *C. finmarchicus* may contribute to more sustainable feed formulations by enabling further reduction of fishmeal content, while supporting fish performance or health. Its application, therefore, represents a practical step toward resource-efficient and resilient aquaculture systems, in line with broader policy goals such as the EU's Farm to Fork strategy and Sustainable Feed for Sustainable Aquaculture [14], the Strategic Guidelines for a More Sustainable and Competitive EU Aquaculture [15], and responsible use of marine resources highlighted by FAO [16]. Together, these frameworks emphasize the need for circular solutions, diversification of feed ingredients, and reduce dependency on traditional fishmeal sources to ensure a more sustainable, secure, and environmentally responsible aquaculture sector.

In a previous study, a novel protein hydrolysate from *C. finmarchicus* was found to increase fish growth rate and muscle development [17]. The present study is a secondary outcome of the same trial, sampling several additional fish aiming to examine the effects of the hydrolysate on health and welfare of European sea bass, based on the scientific hypotheses of providing improving effects [18, 19]. *C. finmarchicus* hydrolysate was benchmarked at 4% feed inclusion against hydrolysates from sardine, tuna, and salmon. Benchmarking a novel ingredient using other marine hydrolysates as controls would yield more relevant results than comparing it to low-grade ingredients, knowing that hydrolysates often have anti-inflammatory and antioxidative properties [7, 20]. Biomarkers for oxidative stress, intestinal health, and inflammatory response were analyzed at the end of the trial to evaluate the effect of the ingredients on fish health indicators.

## 2. Materials and Methods

### 2.1. Test Hydrolysates and Experimental Diets

Four different diets were produced with low fish meal contents, each with an approximate 3%–4% inclusion of a marine hydrolysate, selected as the minimal level to achieve measurable differences in the trial. Different inclusion levels were used to account for the different protein levels in the hydrolysates, providing each diet with the same crude protein level (Table S1–S3).

A novel hydrolysate from *C. finmarchicus* raw material (produced by Calanus AS) was benchmarked using three diets with commercial hydrolysates (supplied by SPAROS) from sardine, tuna fish, and salmon as controls. The composition of the test hydrolysates and the formulations and analyses of the experimental diets are included in the supporting information (Table S1–S3). A detailed description of the production of the experimental diets, and their chemical analyses has been published previously [17]. All diets were formulated as isonitrogenous (48% crude protein), isolipidic (16% crude fat), isoenergetic (21.9 MJ/kg), and with equal amino acid and fatty acid profiles.

### 2.2. Ethical Statement

The feeding trial was performed at the experimental facilities of SPAROS (Olhão, Portugal), registered for experimentation with aquatic species by Direção-Geral de Alimentação e Veterinária (Ministry of Agriculture, Portugal). The experimental protocol was approved by the Animal Welfare Committee - Órgão Responsável pelo Bem-Estar Animal (ORBEA) of SPAROS (Trial reference: CALBASS). Experiments were conducted by FELASA certified scientists and technical staff, in full compliance with ARRIVE guidelines and the European (Directive 2010/63/EU) and Portuguese (Decreto-Lei no. 113/2013, August 7th) legislation on the protection of animals for scientific purposes.

### 2.3. Feeding Trial

**Figure 1:**
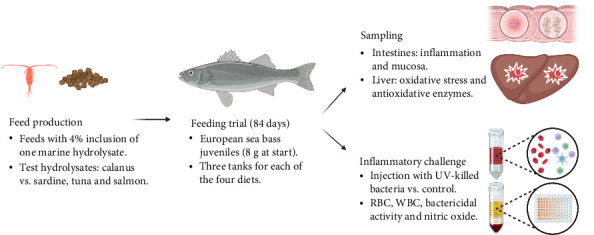
The schematic representation of the feeding trial with European sea bass juveniles, benchmarking the effect of marine protein hydrolysates as functional feed ingredients for health. RBC: red blood cells. UV: ultraviolet radiation. WBC: white blood cells.

Juveniles of European sea bass with an initial body weight of 8.9 ± 0.4 g were fed the experimental diets for 84 days, with triplicate tanks (of 30 fish) for each diet. The fish were sourced from Estação Piloto de Piscicultura de Olhão (EPPO) at the Portuguese Institute of the Sea and Atmosphere (IPMA) and transferred by an authorized carrier to the experimental facilities at SPAROS, which performed the trial. No significant mortality or pathological signs were observed during transport and the fish were kept in sanitary quarantine for 3 weeks before the start of the trial, meanwhile receiving a commercial diet. Fish were grown in 90 L sub-square PVC tanks (30 fish per tank), and only one fish died during the trial. The fish were fed to visual satiety four times a day in indoor tanks with flow-through saltwater and photoperiods of 12 h light and 12 h dark. The water temperature was 23.0 ± 1.4°C, salinity 35.8 ± 0.5 ‰, dissolved oxygen was kept above 5.3 mg/L, and ammonia levels were below the detection limit. A workflow summarizing the feeding trial design is presented in Figure 1.

### 2.4. Fish Sampling

Three fish per replicate tank were euthanized with a lethal dose of anesthesia (AQUI-S, New Zealand) and sampled for specific organs, to assess how dietary inclusion of the test hydrolysates affected oxidative stress and intestinal status using biomarkers and histological samples. The liver was frozen in liquid nitrogen and stored at −80°C until subsequent analysis of oxidative criteria. Part of the distal intestine was preserved in a 10% formalin solution for histology. After dissecting out the posterior intestine, fecal content was collected and stored at −20°C for analysis of calprotectin and mucins. Fish were bulk weighed for growth parameters, described in detail in Bøgwald et al. [17].

### 2.5. Analyses of Antioxidant Status

Liver samples were homogenized with phosphate buffer (0.1 M, pH 7.4) and analyzed for soluble protein concentration, protein carbonyl content, superoxide dismutase (SOD) activity, and catalase (CAT) activity. The Micro BCA Protein Assay Kit (Thermo Scientific, Ref. 23235) was used to determine the soluble protein concentration, with bovine serum albumin as a standard. The protein carbonyl content was determined using a commercial kit from Sigma–Aldrich (Ref. MAK094). SOD activity was measured using the ferricytochrome C method according to McCord and Fridovich [21], and CAT activity was determined by measuring the decrease in hydrogen peroxide concentration at 240 nm according to Aebi [22].

### 2.6. Analyses of Intestinal Status

For assessment of intestinal histomorphology, fragments of the distal intestine were fixed in phosphate-buffered formalin for 24 h and subsequently stored in ethanol (70%) until further processing and sectioning by standard histological techniques. Sections were cut at 3 µm, mounted on glass slides, and stained with hematoxylin and eosin (HE) for histological analysis. The impact of dietary treatments was assessed by light microscopy in terms of villus height (µm, measured from the tip to the base of the villus), villus width (µm), and presence of goblet cells per area of epithelium layer. These measures were made using images obtained with a Nikon Eclipse e200 microscope equipped with a camera and accompanying analysis software (Touptek E3CMOS 3.0 DC). Intestinal permeability was evaluated by measuring the levels of tight-junction protein 2 (zonula occludens-2; ZO-2) in the distal intestine, using a commercial Quantitative Sandwich ELISA kit (MyBioSource, Ref. MBS008988). Intestinal inflammation was evaluated using commercial kits, measuring fecal levels of mucins (CosmoBioCo Ltd, Ref. FFA-MU-K01) and a putative calprotectin fish homolog (MyBioSource, Ref. MBS010625).

### 2.7. Inflammatory Challenge and Analyses

Following the initial feeding trial and all associated samplings, fish from each dietary treatment were fed the experimental diets for one additional week. After this period, fish were subjected to an inflammatory challenge to assess their short-term humoral immune response. To characterize the basal preinflammatory status (T0), three fish per replicate tank (i.e., nine fish per diet) were subjected to a bath containing moderate anesthesia (20 mL/L of AQUI-S, New Zealand) and blood samples were collected by puncture of the caudal vein with a heparinized syringe. Part of the blood sample was used for red blood cell (RBC) and white blood cell (WBC) counts, while the rest was centrifuged at 1 590 × *g* for 10 min. Plasma was transferred to Eppendorf tubes, frozen in liquid nitrogen and stored at −80°C until subsequent analyses of additional humoral parameters. Total RBC and WBC were counted using heparinized (30 units/mL) diluted blood in a Neubauer chamber [23].

After the pre-inflammatory sampling, fish from each replicate tank were intraperitoneally injected with 100 µL of UV-killed *Photobacterium damselae* subsp. *piscicida* (Phdp) (*n* = 12) [24]. Upon injection, fish were redistributed into new tanks according to diet and injection stimuli. Twenty-four hours postinjection (T24), fish injected with Phdp (*n* = 3 per replicate tank) were subjected to moderate anesthesia (20 mL/L of AQUI-S, New Zealand) and a sample of blood was collected by puncture of the caudal vein with a heparinized syringe. The blood was prepared in the same way as before the inflammatory challenge, as described above. Previous research on teleost fish such as European sea bass has demonstrated that significant changes in immune responses are evident within 24 h postchallenge with Phdp [25, 26].

Lysozyme activity was assessed by a turbidimetric assay, following the methodology described by Costas et al. [27]. Briefly, a suspension of *Micrococcus luteus* was prepared (0.5 mg/mL, 0.05 M sodium phosphate buffer, pH 6.2). Fifteen microlitre of plasma was added to a microplate and 250 µL of the above suspension was pipetted to a final volume of 265 µL. The reaction was performed at 25°C and the absorbance (450 nm) was measured after 0.5 and 4.5 min in a Synergy HT microplate reader. Serial diluted, lyophilized hen egg white lysozyme in sodium phosphate buffer (0.05 M, pH 6.2) was used as a standard curve. Nitric oxide (NO) production was measured indirectly based on the Griess reaction described by Chen et al. [28]. Fifty microlitre of plasma was incubated with 100 µL of sulfanilamide (Sigma-Aldrich, 1% in phosphoric acid at 2.5%) for 10 min at room temperature, and afterwards with 100 µL N-naphthylethylene-diamine (Sigma-Aldrich, 0.1% in phosphoric acid at 2.5%) for additional 10 min. Absorbance was read 540 nm in a microplate reader (Synergy HT, Biotek) and nitrite molar concentration was obtained against a sodium nitrite standard curve. Bactericidal activity was measured according to Graham et al. [29], with some modifications by Machado et al. [23]. In short, 10 µL of plasma was added to duplicate wells of a 96-well microplate with Hank's balanced salt solution (HBSS) as positive control. After adding 20 µL of *Vibrio harveyi* (3.13 × 10^6^ CFU/ml) to each well, the plate was incubated for 2.5 h at 25°C. Twenty five microlitre of 3-(4,5-dimethyl-2-yl)-2,5-diphenyl tetrazolium bromide (1 mg/mL, Sigma-Aldrich) was added to each well and the plate was incubated for 10 min at the same temperature. After incubation, the microplates were centrifuged at 2000 × *g* for 10 min and the precipitate was dissolved in 200 µL of dimethyl sulfoxide (DMSO, Sigma-Aldrich). Absorbance of dissolved formazan was read at 560 nm in a Synergy HT microplate reader. Bactericidal activity against *Vibrio harveyi* was expressed as percentage of nonviable bacteria, calculated as the difference between the absorbance of bacteria surviving compared to the absorbance of bacteria from positive controls (100%).

### 2.8. Statistical Analysis

Evaluation criteria are presented in tables or figures with scatter dot plots (dots representing individual data points and a line at the mean for each tank). All data were normally distributed with equal variances (Table S4.1–S4.4. in supporting information). Nested one-way analysis of variance (nested ANOVA) was performed for all the data, and means were compared with the Tukey method. Statistical significance was tested at a 0.05 probability level. All statistical tests were performed with GraphPad Prism (version 10.0.2).

## 3. Results and Discussion

Benchmarking marine hydrolysates on health criteria showed that dietary inclusion of the novel hydrolysate from *C. finmarchicus* improved intestinal health and reduced oxidative stress in European sea bass juveniles, compared to control diets with commercial hydrolysates. Results from the inflammatory challenge, in which the remaining fish were injected with inactivated bacteria, indicated no statistically significant differences on inflammatory response biomarkers. Furthermore, no significant differences were found in intestinal permeability and histomorphometry markers. The trial did not result in any mortality and the fish fully accepted all feeds. Growth parameters have been published in a separate study [17], the results presented and discussed here are, thus, focused solely on health data.

### 3.1. Oxidative Status

**Figure 2:**
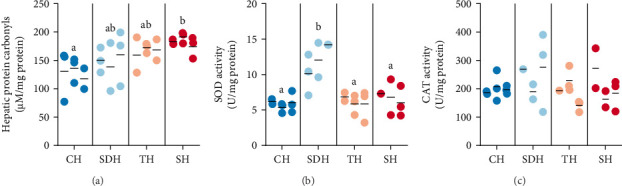
Protein carbonyls and antioxidative enzyme activity as biomarkers of oxidative stress in European sea bass juveniles, measured in liver samples after a feeding trial benchmarking health effects of marine hydrolysates. (A) Hepatic protein carbonyls. (B) Superoxide dismutase activity. (C) Catalase activity. CH: calanus hydrolysate. CAT: catalase. SH: salmon hydrolysate. SDH: sardine hydrolysate. SOD: superoxide dismutase. TH: tuna hydrolysate. U: unit of enzyme activity (µmol/min). Results presented with scatter dot plots (dots representing individual data points and a line at the mean for each tank). Lower case letters (a, b, c) denote statistically significant differences (*p*  < 0.05).

Hepatic protein carbonyl levels were significantly lower in fish fed diets with calanus hydrolysate (CH) compared to the control diet with salmon hydrolysate. At the same time, an intermediate effect was observed for the fish fed sardine and tuna hydrolysates, respectively (Figure 2A). Elevated protein carbonyls in blood and tissues indicate protein oxidation, and this is widely used as a biomarker for oxidative stress, due to its advantages of early formation and relative stability [30]. Increased levels of protein carbonylation have been found in fish species such as rainbow trout (*Oncorhynchus mykiss*), corkwing wrasse (*Symphodus melops*), and eelpout (*Zoarces viviparus*) with oxidative damage due to exposure to oil spills or heavy metals and sewage contamination [31–33]. Oxidative stress is an imbalance of the pro-oxidant/antioxidant homeostasis, causing higher production of reactive oxygen species than the antioxidant defense system can handle. This imbalance can lead to DNA alteration, protein degradation, lipid peroxidation, and cell damage that can lead to apoptosis [34]. In addition to detrimental health implications for living animals, oxidative stress can lead to quality losses of the aquatic food end products [35].

The primary antioxidant enzymes CAT and SOD are part of the crucial first-line defense system, important for preventing and neutralizing the formation of free radicals [36–38]. Their role in health implications have also been found paradoxical, as their activity can promote either health or disease, depending on the physiological context [39]. In this study, hepatic antioxidant enzyme levels were not elevated in fish fed diets with CH (Figure 2B,C), contrary to what could be expected in relation to lower oxidative stress. The most striking result was that the fishfed the sardine hydrolysate diet had a significantly higher SOD activity compared to all other diets (Figure 2B), indicating that something in that diet triggered the system. Low antioxidant enzyme levels can indicate that the red-ox system does not respond sufficiently to increased oxidation, or that cells are exposed to a low degree of oxidative stress [40]. The combination of low levels of oxidation and antioxidant enzymes suggests the presence of other compounds with an antioxidative effect in addition to the endogenous enzymes. Hydrolysates contain bioactive peptides with various activities, of which the antioxidative impact is one of the most documented [41, 42]. Antioxidative peptides reduce the production of reactive oxygen species, and although their exact structure–activity mechanism is not yet fully understood [42], cytoprotective and hepatoprotective effects have been found for protein hydrolysates and antioxidative peptides in several studies [43–46]. The degree of oxidative stress in this study was lowest for European sea bass receiving CH in their diets, suggesting it provides an antioxidative effect that can help maintain the physiological status of fish by preventing cell and tissue damage caused by reactive oxygen species. The antioxidant enzyme activities observed in the current study are in line with previously reported values in juvenile European sea bass under different dietary treatments [47, 48], which suggest that the fish in all treatments maintained redox homeostasis within a physiologically acceptable range.

### 3.2. Intestinal Health

**Figure 3:**
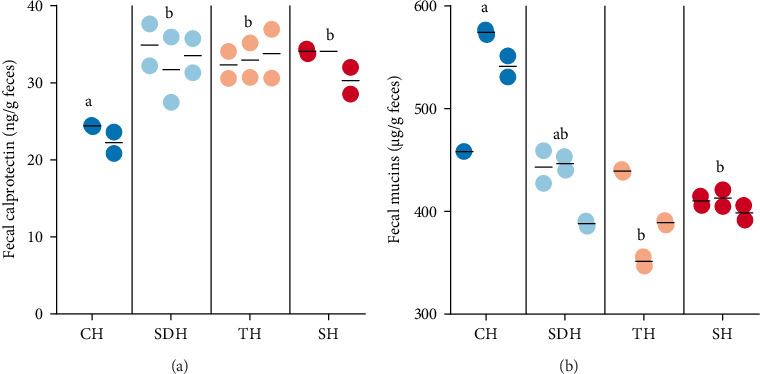
Biomarkers of intestinal inflammation in European sea bass juveniles, measured in feces after a feeding trial that benchmarked health effects of marine hydrolysates as functional ingredients. (A) Fecal calprotectin. (B) Fecal mucins. CH: calanus hydrolysate. SH: salmon hydrolysate. SDH: sardine hydrolysate. TH: tuna hydrolysate. Results presented with scatter dot plots (dots representing individual data points and a line at the mean for each tank). Lower case letters (a, b, c) denote statistically significant differences (*p*  < 0.05).

The intestinal status of European sea bass juveniles was measured using markers of inflammation, permeability, and histomorphometry. The distal intestine was selected as the focus of our assessments, as this region has been shown to be particularly sensitive to dietary protein sources and hydrolysates in teleost fish [49, 50]. In nutritional studies focusing on the gut health of European sea bass (*D. labrax*), measuring villi length and width is a widely accepted method for assessing intestinal morphology [51, 52]. These linear measurements are relatively straightforward to obtain and less susceptible to variability caused by tissue processing or sectioning artifacts, making them reliable indicators of morphological changes due to dietary interventions. No statistically significant differences were found for the intestinal permeability marker tight-junction protein 2, nor for villus height and width or number of goblet cells per area of epithelium layer (Table S5.1, S5.2). However, fish fed diets with CH inclusion showed significantly lower levels of fecal calprotectin-like protein than the control hydrolysate diets (Figure 3A). Although the presence of a calprotectin homolog in fish has been debated [53], it has been used as an indicator of intestinal inflammation since it is a sodium-binding family of proteins involved in many physiological functions, acting as mediators of migration, adhesion, and neutrophil phagocytosis [54]. Elevated calprotectin in feces might, thus, indicate increased migration of neutrophils to the gastrointestinal tissue due to an infection or an inflammatory process. In studies with zebrafish, significant neutrophil and calprotectin responses have been found following *Vibrio cholerae* infection [55]. Elevated levels of S100A10a/A10b, a putative fish analog of the human calprotectin heterodimer S100A8/A9 [56], have also been found in zebrafish infected with *Escherichia coli* [57].

The diet with CH inclusion was also associated with increased fecal mucins, with levels significantly higher than for control diets with tuna and salmon hydrolysates (Figure 3B). Mucins are glycosylated proteins produced by epithelial tissues in most animals and make up the mucus layer of the intestinal barrier. The mucus layer provides an important form of defense through lubrication and mucosal immune responses that prevent bacteria, chemicals, and endotoxins from transferring into the body [58]. Downregulation of intestinal mucins has been found in gilthead seabream infected with the intestinal parasite *Enteromyxum leei* [59], and an increase of mucin-secreting cells, and thus increased mucin levels, have been suggested to be related to improvement in resistance to bacterial infection in European sea bass [60, 61].

The combination of less intestinal inflammation and a higher level of secreted mucins suggests that fish fed diets with CH had better intestinal health than those fed diets with the control hydrolysates. Dietary hydrolysates constituting low molecular weight peptides and metabolites have previously been found to positively influence the intestinal health of European sea bass [62]. Small peptides and metabolites can be transported from the intestinal lumen into the enterocytes by membrane transporters, whose expression of has been found to increase in response to protein content in the lumen of juvenile fish, rendering them highly adaptable to diets with such small peptides [63, 64]. Also, several studies have found that the protein uptake capacity of fish larvae is limited by proteolytic activity instead of the absorptive capacity of the intestinal tract [65–67]. While short peptides could be readily taken up, high amounts of large proteins might interfere with the proteolytic homeostasis and cause inflammation of the intestinal tract. Fish develop their gastrointestinal system as they age, and their underdeveloped digestive tract in early life stages are genetically programed for zooplankton [68], which could explain the especially beneficial effect of dietary zooplankton products in larvae and juvenile stages. A low molecular weight fraction was found to increase skeletal muscle growth in the first-ever study with CH [17], the observed positive effects on intestinal health could also stem from its particularly low molecular weight. CH consists of 87% short peptides and amino acids with molecular weight <1000 Da (Table S6). Since no information about molecular weights for the other hydrolysates was available at the time of this trial, a direct comparison on this basis could not be made here. However, a study on protein hydrolysates, including salmon and mackerel hydrolysates, showed that these had molecular weights several magnitudes higher than the presented values for CH [69]. Therefore, further research is needed to clarify how the molecular weight of different hydrolysates influences intestinal inflammation.

### 3.3. Inflammatory Challenge and Response

*Photobacterium damselae* subsp. *piscicida* (Phdp) is a gram-negative bacterium that causes photobacteriosis, an infection in a wide range of marine animals and one of the most common diseases of farmed European sea bass [70]. In this study, UV-killed Phdp was intraperitoneally injected to trigger an inflammatory status in the fish. Blood cell counts before (T0) and 24 h postinflammation (T24) verified that the pathogen had triggered a reaction, showing decreased RBC and approximately a 2-fold increase of WBC for all diets after the bacterial inoculation (Table 1). No significant between-group differences were found for RBC and WBC responses. Baseline RBC and WBC levels observed at T0 and postchallenge were consistent with ranges, yet in the higher order of what has been reported in European sea bass juveniles exposed to dietary or environmental modulation and bacterial challenges [71–73]. The observed variability in WBC counts, despite the absence of significant changes in innate humoral markers such as lysozyme, has also been reported following stress-induced immune stimulation [74].

The results for the specific inflammatory biomarkers in the blood seemingly displayed an increased response for fish fed diets with CH and salmon hydrolysate postinflammation (Table 1). Still, no statistically significant differences were found for NO, lysozyme, nor the bactericidal activity against *V. harveyi*.

Lysozyme is an antimicrobial enzyme of the innate immune system, where it is involved in numerous defense mechanisms that protect against microbial invasion [75]. NO is toxic to microbial pathogens and is produced by many cell types involved in immunity and inflammation [76], also in the cardiovascular system of teleost fish [77]. Higher levels of NO and lysozyme in the blood are indicative of a stronger inflammatory response. Likewise, increased bactericidal activity against *V. harveyi* would have suggested that the blood serum contained elevated levels of substances involved in the inflammatory response (antibodies, complement proteins, antimicrobial peptides, and so on).

Both dietary inclusion and intraperitoneal injection of protein hydrolysates with short-chain peptides have been shown to induce a stronger immune response, higher resistance against pathogens, and increased survival of many fish species [7, 78]. The lack of statistically significant differences for the inflammatory biomarkers in this study could suggest that too few experimental replicates were tested, that the hydrolysate inclusion levels were too low, or that there simply were no differences in effect among the diets.

## 4. Conclusions

The findings demonstrate that CH can function as a beneficial dietary component for European sea bass, with measurable improvements in oxidative and intestinal health. The significant reduction in hepatic carbonyls indicates that CH can mitigate oxidative stress, suggesting its potential as a natural antioxidant source. Furthermore, the observed decrease in calprotectin levels and increased mucin production indicate enhanced intestinal integrity and reduced inflammation, both key indicators of improved gut health and functionality.

Although the inflammatory challenge with Phdp did not reveal differences between the diets, the outcomes collectively position CH as a promising functional ingredient in aquaculture nutrition. Further investigation into its bioactive components, particularly antioxidative peptides, and the role of protein hydrolysate molecular weight in modulating gut health could provide additional insight into the results of the present study.

## Figures and Tables

**Table 1:** Inflammatory response parameters (*n* = 3) measured in the blood of European sea bass juveniles, before and 24 h after injection with UV-killed *Photobacterium damselae* subsp. *piscicida* (Phdp).

Parameter	CH	SDH	TH	SH
Preinflammation (T0)				
RBC (10^6^/µL)	1.11 ± 0.14	1.03 ± 0.10	1.05 ± 0.03	1.13 ± 0.08
WBC (10^4^/µL)	7.06 ± 2.18	7.56 ± 0.92	6.39 ± 2.61	7.53 ± 1.65
Nitric oxide (µM)	26.9 ± 10.2	30.3 ± 8.1	27.9 ± 7.1	27.7 ± 10.1
Lysozyme (µg/mL)	13.4 ± 2.4	11.2 ± 0.8	10.6 ± 2.0	11.6 ± 0.3
Vibrio inhibition (%)	21.4 ± 2.4	17.3 ± 6.3	13.1 ± 3.7	19.4 ± 5.5
Postinflammation (T24)				
RBC (10^6^/µL)	0.71 ± 0.13	0.64 ± 0.12	0.74 ± 0.13	0.74 ± 0.04
WBC (10^4^/µL)	17.00 ± 3.01	14.17 ± 1.59	15.72 ± 2.55	16.56 ± 3.56
Nitric oxide (µM)	29.6 ± 5.8	23.4 ± 3.0	24.9 ± 3.1	29.3 ± 6.5
Lysozyme (µg/mL)	12.5 ± 1.3	10.2 ± 2.9	9.7 ± 1.7	11.9 ± 1.7
Vibrio inhibition (%)	28.0 ± 2.6	22.6 ± 4.3	26.0 ± 4.1	29.7 ± 1.7

*Note:* T0: Time at start of inflammatory challenge. T24: 24 h after inflammatory challenge start. Statistical significance was tested with no definitive differences among the diets (*p*  < 0.05).

Abbreviations: CH, calanus hydrolysate; RBC, red blood cells; SDH, sardine hydrolysate; SH, salmon hydrolysate; TH, tuna hydrolysate; WBC, white blood cells.

## Data Availability

All the data are available in the article or the supporting information.

## Supporting Information

Supporting Information The supporting information include the composition of test hydrolysates, information on the formulated experimental diets, summaries of ANOVA tests used to test statistical significance, results for intestinal permeability and histology, and molecular weight distribution of calanus hydrolysate. Table S1. Nutrient composition of the test hydrolysates. Table S2. Formulation of experimental diets. Table S3. Analytical composition of experimental diets (as fed basis). Table S4.1–S4.4. Nested ANOVA summaries for all presented results. Table S5.1–S5.2. Intestinal permeability and histomorphometry markers. Table S6. Molecular weight of calanus hydrolysate (CH).
