# Supplementary material for: Dietary Protein Hydrolysate From Calanus finmarchicus Reduces Oxidative Stress and Increases Intestinal Health in European Sea Bass (Dicentrarchus labrax) Juveniles
Source: Aquac Nutr. 2025 Aug 6;2025:5531437. doi: 10.1155/anu/5531437 (PMC12349996; doi:10.1155/anu/5531437)
Supplement: Supporting Information — The supporting information include the composition of test hydrolysates, information on the formulated experimental diets, summaries of ANOVA tests used to test statistical significance, results for intestinal permeability and histology, and molecular weight distribution of calanus hydrolysate. Table S1. Nutrient composition of the test hydrolysates. Table S2. Formulation of experimental diets. Table S3. Analytical composition of experimental diets (as fed basis). Table S4.1–S4.4. Nested ANOVA summaries for all presented results. Table S5.1–S5.2. Intestinal permeability and histomorphometry markers. Table S6. Molecular weight of calanus hydrolysate (CH). [file 5531437.f1.docx]

**Supplementary materials**

The supplementary materials include the composition of test hydrolysates, information on the formulated experimental diets, summaries of ANOVA tests used to test statistical significance, results for intestinal permeability and histology, and molecular weight distribution of calanus hydrolysate.

Table S1. Nutrient composition of the test hydrolysates.

Table S2. Formulation of experimental diets.

Table S3. Analytical composition of experimental diets (as fed basis).

Table S4.1-S4.4. Nested ANOVA summaries for all presented results.

Table S5.1-S5.2. Intestinal permeability and histomorphometry markers.

Table S6. Molecular weight of calanus hydrolysate (CH).

Table S1. Nutrient composition of the test hydrolysates.

| Product form | Liquid | Powder | Powder | Liquid |
| --- | --- | --- | --- | --- |
| Testing Name | CH | SDH | TH | SAH |
| Moisture, % | 46.7 | 3.3 | 3.2 | 51.2 |
| Crude protein, % | 33.6 | 79.5 | 76.3 | 35.0 |
| Crude fat, % | 0.6 | 0.2 | 7.2 | 3.9 |
| Ash, % | 10.9 | 15.6 | 13.1 | 5.1 |
| Phosphorus, % | 0.79 | 0.94 | 0.81 | 1.22 |
| Gross energy, kJ/g | 9.8 | 18.4 | 21.5 | 10.7 |
|  |  |  |  |  |
| Arginine, % | 2.23 | 3.38 | 4.60 | 2.12 |
| Histidine, % | 0.46 | 1.88 | 2.13 | 0.87 |
| Isoleucine, % | 1.21 | 1.40 | 2.20 | 1.42 |
| Leucine, % | 2.07 | 3.11 | 4.19 | 2.29 |
| Lysine, % | 2.26 | 3.57 | 5.35 | 2.44 |
| Threonine, % | 1.23 | 1.73 | 3.02 | 1.42 |
| Valine, % | 1.58 | 2.03 | 3.09 | 1.73 |
| Methionine, % | 0.62 | 0.71 | 1.44 | 0.96 |
| Cysteine, % | 0.33 | 0.30 | 0.55 | 0.37 |
| Phenylalanine, % | 1.08 | 1.50 | 2.82 | 1.12 |
| Tyrosine, % | 1.19 | 0.61 | 0.82 | 1.03 |
| Aspartic acid, % | 2.56 | 4.12 | 6.04 | 2.82 |
| Glutamic acid, % | 3.85 | 7.40 | 9.48 | 3.91 |
| Alanine, % | 2.12 | 5.48 | 6.18 | 2.10 |
| Glycine, % | 2.29 | 9.70 | 10.23 | 2.58 |
| Proline, % | 1.13 | 3.97 | 5.57 | 1.72 |
| Serine, % | 1.11 | 1.88 | 2.88 | 1.36 |
| Taurine, % | 0.56 | 0.51 | 0.29 | 0.26 |

CH: Calanus hydrolysate; SDH: Sardine hydrolysate; TH: Tuna fish hydrolysate; SAH: Salmon hydrolysate.

Table S2. Formulation of the experimental diets.

| Ingredients, % | CH | SDH | TH | SAH |
| --- | --- | --- | --- | --- |
| Fishmeal Super Prime^1^ | 5.00 | 5.00 | 5.00 | 5.00 |
| Fishmeal 60^2^ | 5.00 | 5.00 | 5.00 | 5.00 |
| Calanus hydrolysate (dry basis)^3^ | 4.20 |  |  |  |
| Salmon hydrolysate (dry basis)^3^ |  |  |  | 3.55 |
| Sardine hydrolysate^3^ |  | 2.95 |  |  |
| Tuna hydrolysate^3^ |  |  | 3.25 |  |
| Brewer's yeast^4^ | 2.50 | 2.50 | 2.50 | 2.50 |
| Soy protein concentrate^5^ | 15.00 | 15.00 | 15.00 | 15.00 |
| Wheat gluten^6^ | 8.50 | 8.50 | 8.50 | 8.50 |
| Corn gluten meal^7^ | 15.00 | 15.00 | 15.00 | 15.00 |
| Soybean meal 48^8^ | 16.00 | 16.00 | 16.00 | 16.00 |
| Rapeseed meal^9^ | 7.50 | 7.50 | 7.50 | 7.50 |
| Wheat meal^10^ | 0.80 | 2.35 | 2.15 | 1.95 |
| Whole peas^11^ | 3.50 | 3.50 | 3.50 | 3.50 |
| Vitamin & Mineral premix^12^ | 1.00 | 1.00 | 1.00 | 1.00 |
| Vitamin E50^13^ | 0.05 | 0.05 | 0.05 | 0.05 |
| Betaine HCl^14^ | 0.15 | 0.15 | 0.15 | 0.15 |
| Antioxidant^15^ | 0.20 | 0.20 | 0.20 | 0.20 |
| Monosodium phosphate^16^ | 1.80 | 1.80 | 1.70 | 1.60 |
| DL-Methionine^17^ | 0.20 | 0.20 | 0.20 | 0.20 |
| Soy lecithin^18^ | 1.00 | 1.00 | 1.00 | 1.00 |
| Fish oil^19^ | 6.50 | 6.50 | 6.50 | 6.50 |
| Rapeseed oil^20^ | 6.10 | 5.80 | 5.80 | 5.80 |

^1^ Super Prime: 66.3% CP, 11.5% CF, Pesquera Diamante, Peru; ^2^ COFACO 60: 62.3% crude protein (CP), 8.4% crude fat (CF), COFACO, Portugal; ^3^ Please check details in Table 1; ^4^ Brewer’s yeast: 39% CP, Premix Lda, Portugal; ^5^ Soycomil P: 62% CP, 0.7% CF, ADM, The Netherlands; ^6^ VITEN: 82% CP, 2.1% CF, Roquette, France; ^7^ Corn gluten meal: 61% CP, 6% CF, COPAM, Portugal; ^8^ Dehulled solvent extracted soybean meal: 47% CP, 2.6% CF, CARGILL, Spain; ^9^ Defatted rapeseed meal: 32.7% CP, 4.1% CF, Ribeiro & Sousa Lda, Portugal; ^10^ Wheat meal: 11.7% CP, 1.6% CF, MOLISUR, Spain; ^11^ Yellow peas: 19% CP, 2% CF, Ribeiro e Sousa Lda, Portugal; ^12^ PREMIX Lda, Portugal. Vitamins (IU or mg/kg diet): DL-alpha tocopherol acetate, 100 mg; sodium menadione bisulphate, 25mg; retinyl acetate, 20000 IU; DL-cholecalciferol, 2000 IU; thiamine, 30mg; riboflavin, 30mg; pyridoxine, 20mg; cyanocobalamin, 0.1mg; nicotinic acid, 200mg; folic acid, 15mg; ascorbic acid, 1000mg; inositol, 500mg; biotin, 3mg; calcium pantothenate, 100mg; choline chloride, 1000mg, betaine, 500mg. Minerals (g or mg/kg diet): cobalt carbonate, 0.65mg; copper sulphate, 9mg; ferric sulphate, 6mg; potassium iodide, 0.5mg; manganese oxide, 9.6mg; sodium selenite, 0.01mg; zinc sulphate, 7.5mg; sodium chloride, 400mg; calcium carbonate, 1.86g; excipient wheat middlings; ^13^ ROVIMIX E50, DSM Nutritional Products, Switzerland; ^14^ Beta-Key 95%, ORFFA, The Netherlands; ^15^ VERDILOX PX, KEMIN EUROPE NV, Belgium; ^16^ Bolifor MSP: 24% P, Yara International, Norway; ^17^ Rhodimet NP99, ADISSEO, France; 18 LECICO GmbH, Germany; ^19^ Sopropêche, France; ^20^ JC Coimbra, Portugal. CH: Calanus hydrolysate; SDH: Sardine hydrolysate; TH: Tuna fish hydrolysate; SAH: Salmon hydrolysate.

Table S3. Analytical composition of experimental diets (as fed basis).

|  | CH | SDH | TH | SAH |
| --- | --- | --- | --- | --- |
| Moisture, % | 5.4 ± 0.0 | 5.5 ± 0.0 | 3.3 ± 0.0 | 3.9 ± 0.1 |
| Ash, % | 7.1 ± 0.4 | 6.9 ± 0.1 | 7.1 ± 0.0 | 6.9 ± 0.1 |
| Crude protein, % | 48.4 ± 0.1 | 48.1 ± 0.1 | 48.7 ± 0.0 | 48.5 ± 0.0 |
| Crude fat, % | 16.1 ± 0.1 | 15.8 ± 0.0 | 16.3 ± 0.1 | 16.5 ± 0.1 |
| Gross energy, kJ/g | 21.7 ± 0.0 | 21.8 ± 0.0 | 22.2 ± 0.0 | 22.1 ± 0.0 |
|  |  |  |  |  |
| Arginine, % | 2.42 ± 0.01 | 2.46 ± 0.02 | 2.38 ± 0.01 | 2.58 ± 0.02 |
| Histidine, % | 1.04 ± 0.01 | 0.99 ± 0.01 | 1.12 ± 0.01 | 1.13 ± 0.01 |
| Isoleucine, % | 1.82 ± 0.01 | 1.78 ± 0.01 | 1.85 ± 0.01 | 1.93 ± 0.01 |
| Leucine, % | 4.17 ± 0.02 | 4.11 ± 0.02 | 4.24 ± 0.03 | 4.30 ± 0.01 |
| Lysine, % | 2.13 ± 0.01 | 2.13 ± 0.02 | 2.29 ± 0.01 | 2.23 ± 0.01 |
| Threonine, % | 1.65 ± 0.01 | 1.67 ± 0.01 | 1.76 ± 0.03 | 1.70 ± 0.01 |
| Tryptophan, % | 0.48 ± 0.01 | 0.46 ± 0.01 | 0.49 ± 0.01 | 0.47 ± 0.00 |
| Valine, % | 2.16 ± 0.01 | 2.06 ± 0.01 | 2.24 ± 0.03 | 2.23 ± 0.01 |
| Methionine, % | 0.97 ± 0.06 | 1.03 ± 0.02 | 1.06 ± 0.01 | 1.08 ± 0.01 |
| Cysteine, % | 0.61 ± 0.01 | 0.60 ± 0.02 | 0.62 ± 0.01 | 0.62 ± 0.01 |
| Phenylalanine, % | 2.29 ± 0.01 | 2.31 ± 0.01 | 2.35 ± 0.01 | 2.38 ± 0.02 |
| Tyrosine, % | 1.69 ± 0.03 | 1.70 ± 0.02 | 1.69 ± 0.01 | 1.66 ± 0.01 |
| Aspartic acid, % | 3.60 ± 0.04 | 3.72 ± 0.02 | 3.99 ± 0.05 | 3.84 ± 0.03 |
| Glutamic acid, % | 9.83 ± 0.02 | 9.25 ± 0.05 | 9.70 ± 0.05 | 9.53 ± 0.05 |
| Alanine, % | 2.46 ± 0.01 | 2.47 ± 0.03 | 2.53 ± 0.02 | 2.54 ± 0.03 |
| Glycine, % | 2.08 ± 0.01 | 2.13 ± 0.02 | 2.16 ± 0.01 | 2.13 ± 0.01 |
| Proline, % | 3.19 ± 0.03 | 3.06 ± 0.02 | 3.33 ± 0.01 | 3.23 ± 0.01 |
| Serine, % | 2.18 ± 0.01 | 2.21 ± 0.01 | 2.28 ± 0.02 | 2.24 ± 0.01 |

Values are means ± standard deviation (n=2). CH: Calanus hydrolysate; SDH: Sardine hydrolysate; TH: Tuna fish hydrolysate; SAH: Salmon hydrolysate.

Table S4.1. Summary of nested one-way ANOVA oxidative stress.

| **Nested one-way ANOVA** | HPC | | SOD | | CAT | |
| --- | --- | --- | --- | --- | --- | --- |
| P value | 0.0047 | | 0.0011 | | 0.4987 | |
| P value summary | ** | | ** | | Ns | |
| Significantly different (P < 0.05)? | Yes | | Yes | | No | |
| F, DFn, Dfd | 5.437, 3, 27 | | 15.27, 3, 8 | | 0.8630, 3, 8 | |
|  |  | |  | |  | |
| **Random effects** | SD | Variance | SD | Variance | SD | Variance |
| Variation within subcolumns | 27.68 | 766.1 | 1.955 | 3.822 | 64.95 | 4218 |
| Variation among subcolumn means | 0.000 | 0.000 | 0.000 | 0.000 | 16.26 | 264.5 |
|  |  | |  | |  | |
| **Do the subcolumns differ**  **(within each column)?** |  | |  | |  | |
| Chi-square, df |  | | 7.105e-014, 1 | | 0.05763, 1 | |
| P value |  | | >0.9999 | | 0.8103 | |
| P value summary |  | | ns | | ns | |
| Is there significant difference  between subcolumns (P < 0.05)? | No | | No | | No | |

Table S4.2. Summary of nested one-way ANOVA intestinal health.

| **Nested one-way ANOVA** | Calprotectin | | Mucins | |
| --- | --- | --- | --- | --- |
| P value | 0.0065 | | 0.0165 | |
| P value summary | ** | | * | |
| Significantly different (P < 0.05)? | Yes | | Yes | |
| F, DFn, Dfd | 11.62, 3, 6 | | 6.343, 3, 8 | |
|  |  | |  | |
| **Random effects** | SD | Variance | SD | Variance |
| Variation within subcolumns | 2.937 | 8.625 | 9.799 | 96.02 |
| Variation among subcolumn means | 0.000 | 0.000 | 40.24 | 1619 |
|  |  | |  | |
| **Do the subcolumns differ**  **(within each column)?** |  | |  | |
| Chi-square, df | 0.000, 1 | | 25.09, 1 | |
| P value | >0.9999 | | <0.0001 | |
| P value summary | ns | | **** | |
| Is there significant difference  between subcolumns (P < 0.05)? | No | | Yes | |

Table S4.3. Summary of nested one-way ANOVA inflammatory response, T0.

| **Nested one-way ANOVA** | **RBC (T0)** | | **WBC (T0)** | | **NO (T0)** | | **Lysozyme (T0)** | | ***V. harveyi***  **Inhibition (T0)** | |
| --- | --- | --- | --- | --- | --- | --- | --- | --- | --- | --- |
| P value | 0.4316 | | 0.9998 | | 0.9780 | | 0.2558 | | 0.2152 | |
| P value summary | ns | | Ns | | ns | | ns | | Ns | |
| Significantly different (P < 0.05)? | No | | No | | No | | No | | No | |
| F, DFn, Dfd | 1.024, 3, 8 | | 0.002881, 3, 8 | | 0.06289, 3, 8 | | 1.640, 3, 8 | | 1.857, 3, 8 | |
|  |  | |  | |  | |  | |  | |
| **Random effects** | SD | Variance | SD | Variance | SD | Variance | SD | Variance | SD | Variance |
| Variation within subcolumns | 0.1482 | 0.02198 | 2.710 | 7.346 | 10.12 | 102.4 | 1.871 | 3.501 | 7.835 | 61.39 |
| Variation among subcolumn means | 0.03049 | 0.0009296 | 0.000 | 0.000 | 4.727 | 22.34 | 0.9358 | 0.8756 | 0.000 | 0.000 |
|  |  | |  | |  | |  | |  | |
| **Do the subcolumns differ**  **(within each column)?** |  | |  | |  | |  | |  | |
| Chi-square, df | 0.03129, 1 | | 0.000, 1 | | 0.3990, 1 | | 0.5377, 1 | | 0.000, 1 | |
| P value | 0.8596 | | >0.9999 | | 0.5276 | | 0.4634 | | >0.9999 | |
| P value summary | ns | | Ns | | ns | | ns | | ns | |
| Is there significant difference  between subcolumns (P < 0.05)? | No | | No | | No | | No | | No | |

Table S4.4. Summary of nested one-way ANOVA inflammatory response, T24.

| **Nested one-way ANOVA** | **RBC (T24)** | | **WBC (T24)** | | **NO (T24)** | | **Lysozyme (T24)** | | ***V. harveyi***  **Inhibition (T24)** | |
| --- | --- | --- | --- | --- | --- | --- | --- | --- | --- | --- |
| P value | 0.7106 | | 0.6294 | | 0.3246 | | 0.3220 | | 0.0867 | |
| P value summary | Ns | | Ns | | Ns | | Ns | | Ns | |
| Significantly different (P < 0.05)? | No | | No | | No | | No | | No | |
| F, DFn, Dfd | 0.4714, 3, 8 | | 0.6060, 3, 8 | | 1.353, 3, 8 | | 1.362, 3, 8 | | 3.144, 3, 8 | |
|  |  | |  | |  | |  | |  | |
| **Random effects** | SD | Variance | SD | Variance | SD | Variance | SD | Variance | SD | Variance |
| Variation within subcolumns | 0.1445 | 0.02089 | 2.621 | 6.868 | 7.273 | 52.90 | 2.339 | 5.472 | 5.105 | 26.06 |
| Variation among subcolumn means | 0.07329 | 0.005372 | 2.327 | 5.417 | 0.000 | 0.000 | 1.473 | 2.170 | 0.7375 | 0.5440 |
|  |  | |  | |  | |  | |  | |
| **Do the subcolumns differ**  **(within each column)?** |  | |  | |  | |  | |  | |
| Chi-square, df | 0.9731, 1 | | 5.160, 1 | | 0.000, 1 | | 2.051, 1 | | 0.008041, 1 | |
| P value | 0.3239 | | 0.0231 | | >0.9999 | | 0.1521 | | 0.9285 | |
| P value summary | ns | | * | | ns | | ns | | ns | |
| Is there significant difference  between subcolumns (P < 0.05)? | No | | Yes | | No | | No | | No | |

Table S5.1 Intestinal permeability and histomorphometry markers.

|  | **TJP-2**  ng/g feces | **Villi length**  µm | **Villi width**  µm | **Goblet cells**  number (#) |
| --- | --- | --- | --- | --- |
| **CH** | 16.9 ± 2.6 | 615 ± 39 | 65 ± 5 | 26 ± 4 |
| **SDH** | 17.6 ± 1.2 | 614 ± 36 | 65 ± 4 | 24 ± 3 |
| **TH** | 17.1 ± 1.4 | 682 ± 61 | 62 ± 5 | 22 ± 6 |
| **SH** | 17.9 ± 2.2 | 661 ± 30 | 73 ± 4 | 28 ± 9 |

TJP-2: Tight-junction protein 2.

Table S5.2. Results from each tank on intestinal permeability and histomorphometry markers.

|  | **TJP-2**  ng/g feces | **Villi length**  µm | **Villi width**  µm | **Goblet cells**  number (#) |
| --- | --- | --- | --- | --- |
| **CH Tank1** | 15.13 | 569.92 | 67.93 | 30 |
| **CH Tank2** | 19.86 | 640.64 | 68.44 | 22 |
| **CH Tank3** | 15.83 | 634.13 | 59.25 | 25 |
| **SDH Tank1** | 18.53 | 635.02 | 64.14 | 23 |
| **SDH Tank2** | 16.30 | 572.65 | 70.10 | 22 |
| **SDH Tank3** | 18.09 | 634.04 | 61.37 | 28 |
| **TH Tank1** | 17.64 | 618.45 | 64.04 | 15 |
| **TH Tank2** | 17.94 | 689.40 | 65.21 | 25 |
| **TH Tank3** | 15.38 | 739.59 | 56.75 | 27 |
| **SH Tank1** | 16.71 | 646.05 | 77.16 | 21 |
| **SH Tank2** | 16.64 | 695.32 | 70.65 | 25 |
| **SH Tank3** | 20.41 | 641.28 | 70.77 | 37 |

TJP-2: Tight-junction protein 2.

Table S6. Molecular weight of calanus hydrolysate (CH).

| **Molecular weight** | **% protein** |
| --- | --- |
| < 20 000 | 0.1 |
| 6 000 – 20 000 | 0.6 |
| 1 000 – 6 000 | 12.2 |
| 200 – 1000 | 34.7 |
| < 200 | 52.5 |
